# Use of a Novel Manikin for Neonatal Resuscitation Ventilation Training

**DOI:** 10.3390/children9030364

**Published:** 2022-03-04

**Authors:** Catherine Chang, Jeffrey Perlman, Erika Abramson

**Affiliations:** Department of Pediatrics, Weill Cornell Medicine, New York, NY 10065, USA; jmp2007@med.cornell.edu (J.P.); err9009@med.cornell.edu (E.A.)

**Keywords:** neonatal resuscitation, neonatal manikin, bag-mask ventilation, ventilation corrective steps

## Abstract

All providers who attend deliveries independently should be well versed in the performance of effective ventilation, assessment of the quality of ventilation/interventions supplied and able to troubleshoot in situations where these may be ineffective. A novel manikin serves as a unique ventilation-focused training tool to practice these clinical skills and decision-making. The data generated by the manikin, with the aid of a facilitator, may be used for formative and summative feedback on an individual level or curricular development on a larger level. This communication describes the importance of focused ventilation training for front-line providers and illustrates how this manikin can be incorporated into an individualized ventilation training program.

## 1. Introduction

Effective bag-mask ventilation (BMV) is the foundation of successful neonatal resuscitation [1]. Importantly, it involves the use of appropriate technique as well as the timely performance of ventilation corrective steps when indicated, i.e., mask adjustment, reposition, suction, opening the mouth, increase pressure and alternative airway (MRSOPA). Effective ventilation is tightly linked to an improvement in heart rate (HR). Taken together, an assessment of HR and chest rise can indicate to the provider(s) whether effective BMV is being employed. In newborns born apneic, the prompt delivery of effective BMV is critical to survival and can prevent the need for more intensive resuscitation (including chest compressions and medication administration) [2,3]. All providers who attend deliveries should be well versed in the performance and assessment of this life saving skill [1].

At many institutions, including our own, pediatric residents and advanced practice providers (APPs) (specially trained delivery room nurses, physician assistants (PAs), nurse practitioners (NPs) and hospitalists) attend low- to moderate-risk deliveries independently as first responders. In this capacity, they are responsible for performing the initial steps, assessing the newborn, calling for additional help and promptly initiating effective BMV (including performance of the ventilation corrective steps as needed) while awaiting the arrival of the rest of the neonatal advanced practice team. Delays or ineffective performance of these steps may result in worsening respiratory depression, leading to the need for a more intensive resuscitation [2].

Delivery room experience and proficiency of performing bag-mask ventilation amongst frontline providers may be variable. In a study that observed pediatric resident performance of neonatal resuscitation in the delivery room, only 54% of residents competently performed the MRSOPA progression when ventilation corrective steps were required [4]. Specifically, repositioning of the mask/airway (“M” “R”) was performed competently 55% of the time, while suctioning/opening the mouth (“S” “O”) was performed competently 90% of the time it was required. No residents performed the increasing pressure (“P”) step competently when it was required. There is limited data on BMV performance by APPs.

## 2. Use of Simulation in Neonatal Resuscitation Training

Simulation is often utilized as the primary modality for the training and assessment of competency prior to independent attendance at deliveries, though the format varies from institution to institution. Training curricula and refreshers targeted toward front-line level providers often cover BMV and some ventilation corrective steps. However, it is often difficult to assess the ability of an individual to perform the ventilation corrective steps systematically and effectively on their own. Rather, groups of providers often participate in neonatal resuscitation simulations as a team and/or are assessed on the performance of the entire NRP algorithm, focusing largely on advanced skills, such as intubation, line placement, and chest compressions [5,6,7,8,9]. This is rarely reflective of real-life neonatal resuscitation team constituency or role-specific responsibilities, particularly in the case of pediatric residents. Validated tools for assessing individuals also exist, though they, likewise, do not capture the details of performance of the ventilation corrective steps for which the front-line level provider is primarily responsible [10,11].

With high costs and time constraints limiting instructor availability, there has also been increasing attention on automated skills training, where learners can independently practice skills on a simulator without an instructor present. This has proven effective in training the specific skills of BMV and cardiac compressions [12,13,14]. However, performance of these skills does not occur in isolation in the context of resuscitation. While the effective delivery of these skills is important to the overall quality of the resuscitation, providers must also be able to identify when these skills are needed, recognize and troubleshoot problems in real time and ably execute them in situations of high stress. A study of delivery room resuscitation performance by pediatric residents demonstrated a decline in competent performance and self-awareness when resuscitation required the recognition that BMV was needed, the performance of BMV or the performance of the ventilation corrective steps [4].

**Figure 1:**
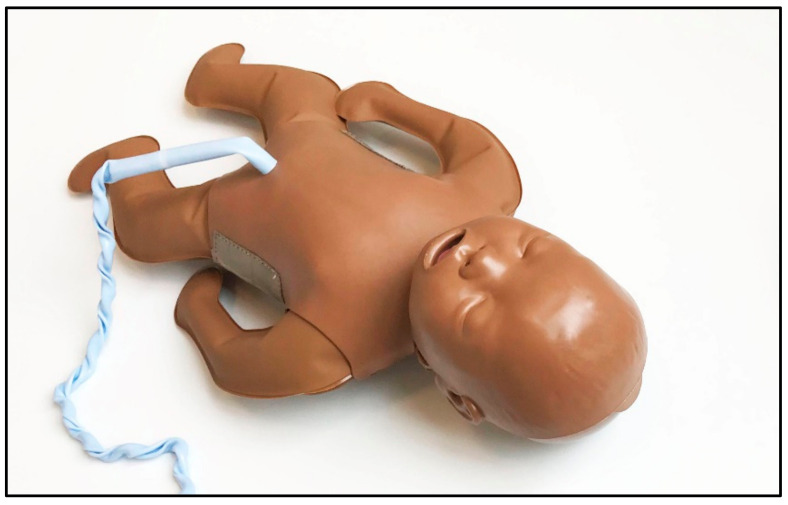
NeoNatalie Live manikin.

The NeoNatalie Live is a novel manikin that automatically measures BMV performance and has the option of annotating the performance of critical resuscitation tasks with facilitator input (Figure 1). These can be utilized to objectively describe learner performance of the initial BMV steps and performance of ventilation corrective steps. In this way, this ventilation-focused manikin may serve as a more comprehensive training and assessment tool in preparing providers for independent delivery room attendance and performance of the initial portion of the neonatal resuscitation program (NRP) algorithm.

The use of the NeoNatalie Live in “individual skill training” mode has been previously described in low- and well-resourced settings [12,15]. Utilization of this mode in recurrent booster or low dose, high frequency training has been shown to improve ventilation performance and promote skill retention among users. However, the use of this manikin in its “scenario training” mode has not been described.

The objective of this report is twofold: first, to describe the use of the NeoNatalie Live’s “scenario training” mode in facilitated sessions with an instructor, its capabilities and objective data outputs and second, to illustrate this manikin’s use in a neonatal resuscitation (NRP) refresher course and how it can be used to drive learner feedback, evaluation and curricular development.

## 3. General Manikin Description

The NeoNatalie Live is a second-generation newborn simulator (Laerdal Medical, Stavanger, Norway). This manikin automatically detects head position (i.e., whether the airway is aligned in the optimal “sniffing” position), mask seal, ventilation attempts, ventilation pressure and ventilation rate. A valve controlling the opening of the airway simulates “normal” or “low” lung compliance, which can be chosen by a facilitator at the start of and during a simulation scenario. Importantly, the manikin also provides a heart rate response to the quality and length of ventilation provided. This data is automatically downloaded to an integrated tablet-based application.

The manikin provides four different case scenarios of varying difficulty based on “lung compliance” (normal/low) and initial heart rate (normal/low). In the “normal compliance” mode, ventilation pressures of 15–20 cmH_2_O will result in chest rise, leading, in turn, to increasing heart rate. In the “low compliance” mode, ventilation at higher ventilation pressures (~30 cmH_2_O) are required to initiate chest rise. Sustained, effective delivery of these higher pressures with the head/neck aligned in the appropriate sniffing position and a good mask seal against the face results in a gradual improvement in chest rise and an increase in heart rate.

The NeoNatalie Live manikin is primarily designed for airway simulation and is optimized for education related to the initial portion of the NRP algorithm, BMV and the ventilation corrective steps. Learners are unable to place an advanced airway, cannulate the umbilical cord or obtain realistic feedback with chest compressions.

## 4. Modes of Training

### 4.1. Individual Skill Training Mode

The scenarios on the NeoNatalie Live can be practiced in either an individual or scenario training mode. In the low-resource setting, this manikin has primarily been used in the individual mode for the independent practice of BMV [12,16]. In this mode, learners may choose a scenario and practice ventilation based on prompts on the tablet application. Specific feedback on the ventilation performance (related to airway positioning, rate and pressure delivered) are provided to the user by the application during and after a practice session.

### 4.2. Scenario Training Mode

The addition of a facilitator broadens the training beyond the practice of isolated ventilation technique into the global management of an apneic newborn and the need for the effective delivery of BMV in the “scenario training” mode. The expanded benefits to the learner(s) are manifold and include the additional assessment of other important clinical and cognitive skills, such as the performance of the initial steps, requesting additional help, asking for heart rate, etc. Second, it facilitates modulating the complexity of scenarios, allowing a targeted and increased range of learning objectives. For example, a facilitator may manually “close” the airway, not permitting chest rise to occur (or HR to increase) to challenge the learner and see if they perform the ventilation corrective steps correctly. An additional benefit includes the ability to explore the provider assessment of the clinical situation and decision-making, providing additional perspectives for more nuanced feedback.

**Figure 2:**
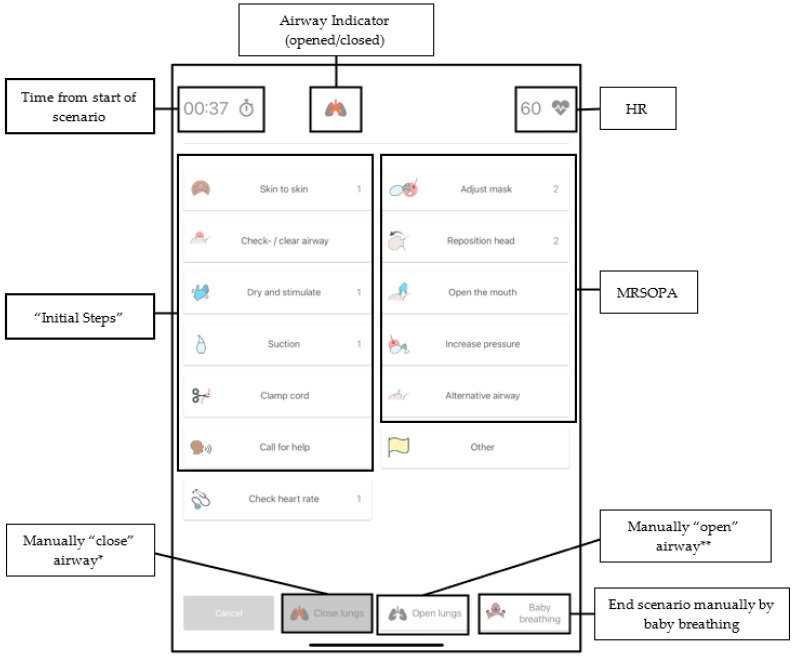
Screenshot of the tablet application. Each button denotes a resuscitation step that may be performed. The facilitator taps each button as the learner performs a particular step in real time, creating a detailed timeline of the performance. (Left column): “initial steps” (airway clearance, dry/stimulate, suction, call for help, etc). (Right column): ventilation corrective steps (MRSOPA) * When the airway is “closed”, no chest rise will occur, regardless of interventions/ventilation provided. ** When the airway is “open”, chest rise will occur if head/airway are in the appropriate sniffing position, there is a good seal and the required ventilation pressure is provided. Pressing this button overrides the minimum pressure required to move the chest (dictated by the scenario selected prior to the start of the scenario). HR = heart rate, MRSOPA = ventilation corrective steps of mask adjustment, reposition airway, suction, open the mouth, increase pressure and consideration of an alternative airway.

In the scenario training mode, the learner does not interact or receive feedback from the tablet during the simulation scenario as in the individual skill training mode. Rather, the tablet is used by a facilitator during the scenario to record the performance of resuscitation steps by a single learner or groups of learners in real time, increasing the ease of data recording. The tablet displays a performance checklist including the components of the “initial steps” (warm, stimulation and suctioning) as well as each of the ventilation corrective steps of MRSOPA, with the “A” being a “consideration of an alternative airway”, as the manikin cannot be intubated (Figure 2). Instructors have the option of “closing the airway” to prevent chest rise.

**Figure 3:**
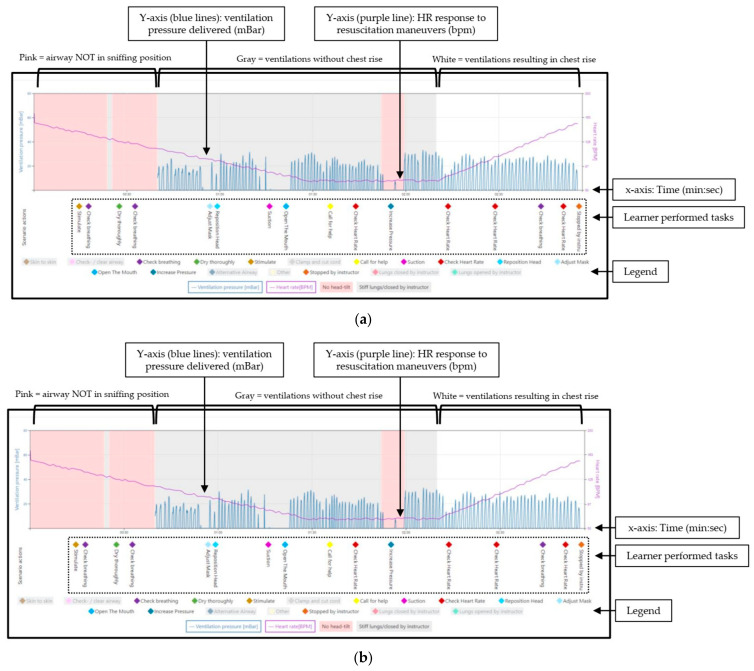
Neonatal resuscitation performance captured by the NeoNatalie Live manikin and tablet application. (**a**) Example of provider baseline performance before the facilitated debriefing with an instructor. (**b**) Example of provider performance after the facilitated debriefing with an instructor. Note that the HR (purple line) decreases steadily while there is no chest rise (gray background). Once effective ventilation is provided and chest rise occurs, the airway is opened (white background) and the HR increases.

After completion of the scenario, learner actions, along with ventilation data from the manikin, are automatically uploaded to an online database and integrated to create a timeline of the resuscitation performance (Figure 3, Table 1).

## 5. Illustration of NeoNatalie Live Use in Provider Training

At our institution, all front-line delivery room providers participate in the American Academy of Pediatrics (AAP) NRP Provider Course training every two years and have active eCards in the “advanced” provider pathway outlined by the NRP [17]. To address knowledge and skill degradation over time, an institutional NRP refresher training course has been created incorporating the use of the NeoNatalie Live manikin. In this course, providers meet individually with an NRP faculty instructor to practice the NRP algorithm and assess competence prior to independent attendance at deliveries. They are asked to review the algorithm, equipment preparation, the initial steps, BMV and the ventilation corrective steps to update knowledge prior to the in-person training session.

### 5.1. Orientation to the Learning Session

At the beginning of the refresher course, the learner is (re)orientated to the manikin and simulated resuscitation area. They are informed of the limitations of the manikin (such as the inability to provide external wetness and that the mouth is fixed in the “open” position). They are asked to suspend disbelief and perform each of the steps of resuscitation despite the manikin’s appearance. To best quantify performance, they are also asked to vocalize each performance step in real time (e.g., “please say you are suctioning when you perform that step. This helps the instructor understand the providers’ intentions and thought processes”). A learner is marked as having completed a task only if they both verbalized what they did and performed the task correctly.

### 5.2. Simulation and Feedback

An initial simulation is performed to establish baseline competency and identify areas to focus on during the debriefing (Figure 3a). During this baseline simulation, the “low lung compliance” scenario is utilized. Learners are presented with a limp/apneic baby after delivery. An ideal performance includes the efficient completion of the initial steps, reassessment that the baby remains limp/apneic, initiation of BMV within one minute of birth (at a rate of 40–60 breaths/minute and pressures of 20–25 cmH_2_O), calling for additional help from the advanced neonatal resuscitation team and asking for a HR. In this “low lung compliance” case, the recommended starting ventilation pressure will not be sufficient to result in chest rise. Additionally, if asked, the learner will be given a declining HR, providing further evidence of inadequate ventilation. Given the lack of chest rise and declining HR, the learner should systematically go through the steps of MRSOP(A), culminating in an increased ventilation pressure, which will result in chest rise and HR increase. If the learner inadvertently provides a pressure of ≥30 cmH_2_O prior to intentionally performing the “pressure increase” step of MRSOPA, the instructor has the option to manually close the airway to encourage performance of the corrective steps.

Following the baseline simulation, a facilitated debriefing is performed with the instructor, with direct feedback of the BMV technique and practice of the ventilation corrective steps. Learner assessments and decision-making are explored. The same simulation scenario is then repeated after debriefing to solidify concepts and determine readiness to attend deliveries independently.

### 5.3. Example of Provider Performance Pre-/Post- Facilitated Debriefing

Figure 3a,b are representative samples of a single provider’s baseline performance and performance after facilitated debriefing and skills practice (respectively). At baseline (Figure 3a), this learner provided initial ventilation pressures that exceeded 30 cmH_2_O, resulting in brief opening of the airway (white area between the gray). The airway was manually closed by the instructor (here denoted by “Other” label), with a return to a gray background. This is done to encourage learner assessment of ineffective BMV and performance of the ventilation corrective steps. This learner did not perform the “initial steps,” performed the ventilation corrective steps out of order and failed to intentionally perform the “pressure increase” step. During the debriefing, the learner stated he/she was unaware of the delivered pressures and had forgotten the “pressure increase” step. On repeat simulation after debriefing and practice (Figure 3b), the learner performed the initial steps, began BMV with the appropriate starting peak inspiratory pressures (which in this scenario was inadequate and did not result in chest movement, indicated as a gray background), then performed the corrective steps in the appropriate order (increasing pressure only when intended as the final step of the MRSOP progression, resulting in a white background) and used an increasing HR as a marker for effective ventilation.

## 6. Discussion

Neonatal resuscitation training curricula should be role-specific and reflect expectations for individual providers at local institutions. For most front-line providers, mastering performance of the initial steps, initiating effective BMV, calling for help from an advanced neonatal resuscitation team and working through the ventilation corrective steps should be prioritized. The NeoNatalie Live is a sophisticated tool for improving the performance of this foundational portion of the NRP algorithm.

In individual training mode, this manikin has been utilized for automated, independent training of the delivery of effective BMV without an instructor. In the broader scenario training mode with a facilitator, learners are challenged with real life delivery room scenarios they may encounter. The ability to control chest rise and the corresponding effect on HR is a unique and important feature of this mode on the NeoNatalie Live that allows instructors to modulate the difficulty and learning objectives. Closing the airway with a declining HR presents the learner with a stressful situation where the newborn is not responding immediately to routine measures. This serves as a critical learning experience and opportunity to practice clinical assessment, decision-making and the execution of interventions. Repeated practice may also improve provider comfort in managing such high-pressure scenarios independently.

The performance reports generated in the scenario training mode can be used in a variety of ways. On the individual level, they can be reviewed during the debriefing to augment instructor-provided formative feedback and coaching on performance. Long term, they can be used as summative feedback for learners, tracking their performance progress over time.

On a larger level, these performance reports may also help define the progression from novice to mastery when learning this critical portion of the NRP algorithm. This may inform the approach to neonatal resuscitation education as well as lend insight into defining competency for independent practice. The availability of these quantitative metrics also opens possibilities for rigorous exploration of educational methods in neonatal resuscitation training. Future studies can more granularly describe how providers work through the steps of the algorithm to understand how people learn, areas to focus on during training and how to adjust the curricula to train people more effectively on the algorithm.

## Figures and Tables

**Table 1:** Description of neonatal resuscitation performance (Figure 3a,b).

Background Colors and Meanings			
Background Color	Airway Valve	Chest Rise Visible When BMV Delivered?	Clinical Scenario Represented
Pink	Closed	No	Manikin’s airway has not been placed in the sniffing position by the learner
Gray	Closed	No	Insufficient peak inspiratory pressure delivered (dependent on case selected) or Airway manually closed by instructor
White	Open	Yes	Effective BMV applied (good seal, appropriate airway position and sufficient pressure delivered per case scenario)
**Other Labels**			
**Label**	**Description**		
*X*-axis	Timeline of the scenario (minute:second format)		
Left *Y*-axis (pressure)	Measured delivered ventilation pressure appears as blue peaks. Each peak represents one delivered breath. Pressure is measured in mBar (1 mBar is approximately equal to 1 cmH_2_O).		
Right *Y*-axis (HR)	Modeled manikin HR response appears as a purple line.		

BMV = bag mask ventilation, HR = heart rate.
